# Trade-offs in mechanical performance influence the diversity of fangs, stingers, and spines

**DOI:** 10.1126/sciadv.aec5395

**Published:** 2026-07-08

**Authors:** Philip S. L. Anderson, Bingyang Zhang, Kehan Pan, Bradley Scott, Abby Weber

**Affiliations:** Department of Evolution, Ecology and Behavior, University of Illinois, Urbana, IL 61801, USA.

## Abstract

Puncturing structures such as fangs, stingers, and spines have evolved convergently across all biological realms. Although superficially similar in form, the diversity of these features calls into question whether simple geometric laws can fully describe their evolution. We examine biological puncture tools through the lens of engineering to evaluate how mechanical principles influence their diversity in nature. Plotting biological puncture tools from more than 140 organisms onto mechanically informed performance landscapes shows that tool diversity is heavily influenced by trade-offs between buckling resistance and puncture efficiency. Where an organism falls on this trade-off is partly related to biological function but shows little relation to the replaceability of the tool. Our results highlight multiple avenues for the evolutionary adaptation of biological puncture systems to constraints imposed by physical laws.

## Teaser

Fangs, spines, and stingers are incredibly diverse in form and function despite potential constraints imposed by physical laws.

## INTRODUCTION

Puncture, defined as the use of a sharpened tool to penetrate a target while the target remains intact (*1*), is widespread in biology. Examples span several orders of magnitude in size (from the stinging cells of jellyfish to the horns of rhinoceros) and speed (passive cactus spines to the sharpened mandibles of some trap-jaw ants). These systems are used for a variety of functional purposes, including prey capture (*2*–*3*), injection (*4*–*5*), defense (*6*–*8*), and even reproduction (*9*–*10*). Regardless of these vast functional differences, it takes nothing more than a cursory glance to note that puncture tools tend to follow a standard morphology: long and slender. Reconciling this seemingly simple morphology with such a vast diversity of functions is a challenge. Most studies on functional morphology are able to focus on a particular type of structure, such as shells, or teeth or limbs, where there is at least the baseline of morphological variety within the same structure. Puncture tools run the gamut from teeth, to claws, to integumentary features to reproductive structures. Addressing the diversity of puncture tools requires examining them at their most basic level, tools used to create fracture and space. Here, we use our understanding of fracture mechanics to explore the relationship between shape and performance across a broad range of biological puncture tools representing most major taxonomic and functional groups, to explore potential mechanical constraints common across biology.

Numerous previous studies have examined the relationship between puncture tool shape and performance in a range of systems. The relationship between tool morphology and fracture performance has been quantified in groups such as mammalian dentitions (*11*–*15*), viper fangs (*16*, *17*), porcupine quills (*6*), fish spines (*18*), cone snails (*19*), Hymenoptera stingers (*20*, *21*), and various plant structures (*22*, *23*). Other studies have examined how specific target materials respond to puncture (*24*–*26*) as well as how deformation of the tool itself may alter puncture performance (*27*–*31*). Even work in the archeological literature has examined the influence of tool shape on projectile puncture (*32*, *33*). While there is general consensus concerning “sharp” tools showing higher puncture performance, what constitutes sharp is not always clear. Experimental work has shown that different aspects of tip shape can influence performance to different degrees (*16*). Furthermore, while the tip is often most important for initiating fracture, actual insertion of the tool will be influenced by the overall shape of the tool, with a complex interplay between fracture, deformation, and friction controlling performance (*34*). Recently, work on dynamic puncture, defined as puncture occurring at more than 1 m/s, has illustrated that speed can alter the relationship between tool shape and performance (*35*).

Several recent studies have used geometric laws in an attempt to describe the variation of puncture tools across nature (*36*–*38*). While these methods offer powerful insights into developmental growth patterns and allow for reconstructions of broken or fossil forms (*37*), there are limitations in how these studies have related the morphological diversity to mechanical performance (*36*, *38*). One issue is that these studies are focused solely on slenderness of the tools and assume biological tools are circular in cross section, when most are actually elliptical and some are quite flat. However, a recent study on carnivoran canines, which does take both the slenderness and flatness into account (*15*), illustrates the importance of this measure in understanding potential function. Flatness will be of even greater importance when examining puncture tools across phyla. Perhaps more critical is that performance is usually measured in terms of forces and not energy. Recent experimental and theoretical work on puncture mechanics has illustrated that energy is often the limiting factor on puncture performance, not absolute force (*34*, *39*–*40*). This is in line with previous work on fracture mechanics in compliant, displacement-limited materials such as soft biological tissues like skin or muscle, which generally require less force but no less energy to successfully fracture (*41*).

Here, we synthesize our understanding of fracture mechanics and biological morphology to evaluate how puncture energetics may influence the diversity of biological puncture systems seen in nature. We evaluate the performance of simulated puncture tools spanning a range of slenderness (taper) and cross-sectional shape (roundness) and create puncture performance landscapes. Performance landscapes help visualize the form-function relationship in multivariate data (*42*) and can explain morphological diversity through the lens of mechanical performance (*43*). We create landscapes based on individual performance variables as well as “combined” landscapes derived from multiple performance metrics. When biological puncture tools (including vertebrates, invertebrates, and plants) are plotted onto this performance space, they cluster in the combined landscape in a way that illustrates fundamental trade-offs between tool robustness and puncture ability.

## RESULTS

### Puncture tool performance landscapes

**Fig. 1.**
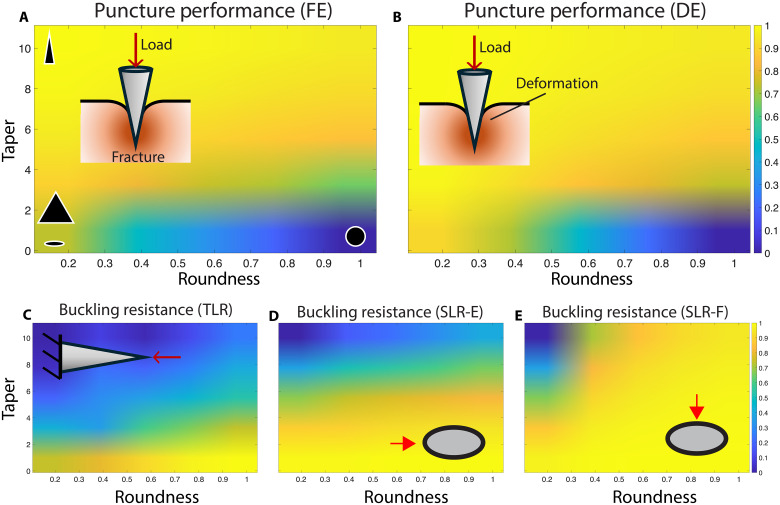
Performance landscapes for two puncture metrics and three buckling metrics. The morphospace is based on the taper (*y* axis) and roundness (*x* axis) of tools, and performance is derived from FEAs on a series of cone shapes across the morphospace. In all plots, performance is normalized to be between 0 and 1 with yellow representing high performance and blue low performance. (**A**) Performance landscape of puncture performance measured as energy expended to create fracture surface (FE). (**B**) Performance landscape of puncture performance measured as energy lost to material deformation (DE). (**C**) Performance landscape of tool buckling resistance based on loads applied at the tip of the tool (TLR). (**D**) Performance landscape of tool buckling resistance based on loads applied to the side of the tool parallel to the long axis of the base (the edge; SLR-E). (**E**) Performance landscape of tool buckling resistance based on loads applied to the side of the tool parallel to the short axis of the base (the flat; SLR-F).

Puncture performance landscapes illustrate a clear trade-off between the buckling resistance of puncture tools and their ability to puncture materials efficiently (Fig. 1). These landscapes are derived from finite element analyses (FEAs) on theoretical cone shapes that vary in both taper (height/base width) and roundness (the ratio of the minor and major axis of the base cross section where low roundness is elliptical and high roundness is circular). Buckling resistance is measured under tip-loading resistance (TLR) and side-loading resistance of the cone from both the edge (SLR-E) and flat (SLR-F) sides. Puncture efficiency is measured as energy required to create fracture surface (FE) and energy lost due to target material deformation (DE).

In general, shapes with high buckling resistance (represented by TLR and SLR) tend to be in the lower right (low taper, high roundness), although the boundaries between low and high performance shift based on the type of buckling (Fig. 1). When buckling forces are applied at the tip, there is a smaller area of high performance at the lowest values of taper with a slightly larger region of high performance at high roundness. On the other hand, when a buckling load is applied to the flatter side, most tools perform equally well with the exception of a small region of low performance in the upper left corner (higher taper and low roundness). Buckling loads applied to the narrower side of the tool show a pattern in between the others, with relatively equal regions of high and low performance.

While the performance landscapes based on the two measures of high puncture efficiency (FE and DE) appear mostly similar, there is a slight difference between them. In general, both landscapes show a large area of high-performance space encompassing low taper and low roundness forms as well as high taper and high roundness forms. However, a closer look shows that when DE is used, the region of high performance is larger, especially at lower roundness.

**Fig. 2.**
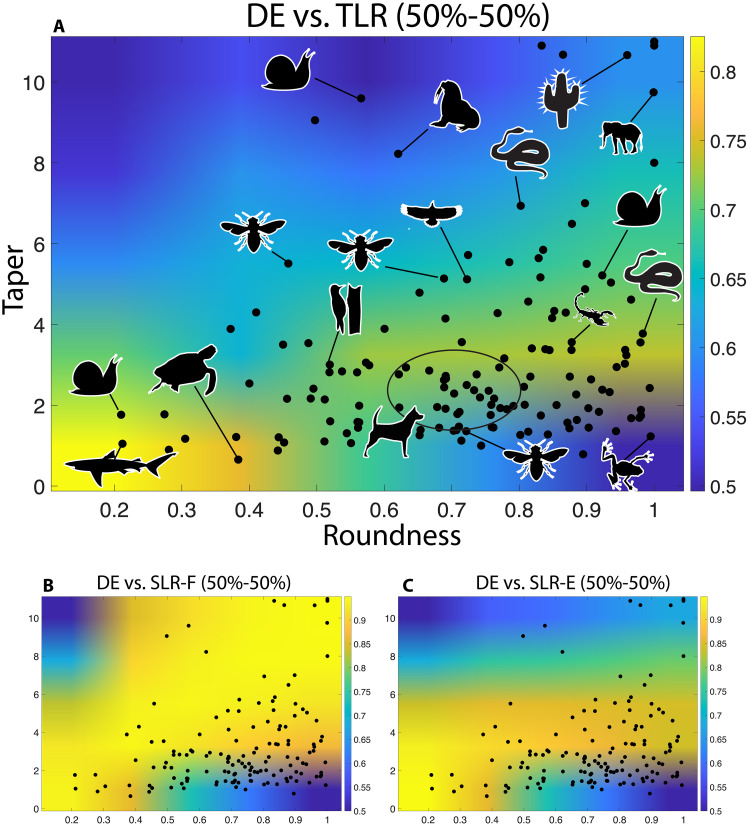
Combined performance landscapes based on energy lost to material deformation and various buckling metrics illustrate trade-offs in biological puncture systems between resistance to buckling and puncture efficiency. Gradient colors as in Fig. 1. Black dots represent 143 biological puncture tools plotted into the morphospace based on their taper and roundness. (**A**) Combined plot of DE and TLR. Silhouettes illustrate the placement of example taxa for reference. Note that the ellipse at the bottom associated with the dog silhouette represents the spread of carnivorans on the plot. (**B**) Combined plot of DE and SLR-F (force applied to the flat side of the tool). (**C**) Combined plot of DE and SLR-E (force applied to the edge side of the tool).

**Fig. 3.**
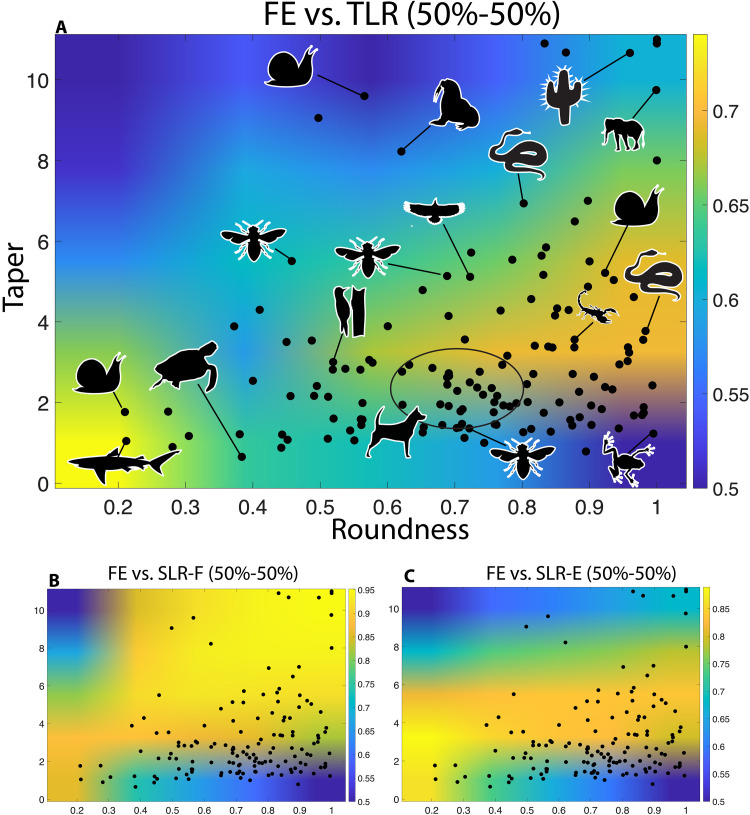
Combined performance landscapes based on energy to create new surface (FE) and various buckling metrics. (**A**) Combined plot of FE and TLR. Silhouettes as in Fig. 2. (**B**) Combined plot of FE and SLR-F. (**C**) Combined plot of FE and SLR-E.

Together, these plots illustrate a potential trade-off between tool robustness and puncture ability that agrees with previous assessments on mammalian taxa (*28*). Our results here illustrate that it may be a broader pattern across biology. The trade-off is brought into sharp focus when puncture and buckling performance metrics are plotted onto combined performance landscapes (Figs. 2 and 3). Following established methods for stacked performance landscapes (*43*), we created landscapes that combine buckling resistance and puncture efficiency by weighting the relative importance of each performance metric. Figures 2 and 3 show all six combined performance landscapes created from the two puncture and three buckling metrics. The main overall trend is a ridge of high performance that extends from the lower left corner (low taper and low roundness) across and up toward a region of high roundness and mid-low taper. However, the extent and placement of this region vary depending on the metrics examined.

When TLR is combined with either DE or FE, the landscapes look almost identical (Figs. 2A and 3A). However, there is a minor trade-off associated with the different ways of measuring puncture efficiency. When FE is used, there is higher performance at high roundness, and when DE is used, there is higher performance at low roundness, similar to what is seen in the single metric landscapes. These results make sense. DE is specifically a measure of the energy lost due to deforming the target during insertion, flattened tools (low roundness) will minimize deformation, while FE is a measure of the energy to create new surface, and conical tools (high roundness) have less surface area relative to volume.

Figures 2 (B and C) and 3 (B and C) show DE and FE each combined with both SLR metrics (loading from the flat and edge sides). In all of these, the region of high performance is larger than with tip loading. It should be remembered that these are all comparative measures within each plot, meaning that we should not attempt to equate the performance values between plots. What the expanded region of high performance likely indicates is a broader range of tool shapes with equivalent performance for these combinations of metrics.

**Fig. 4.**
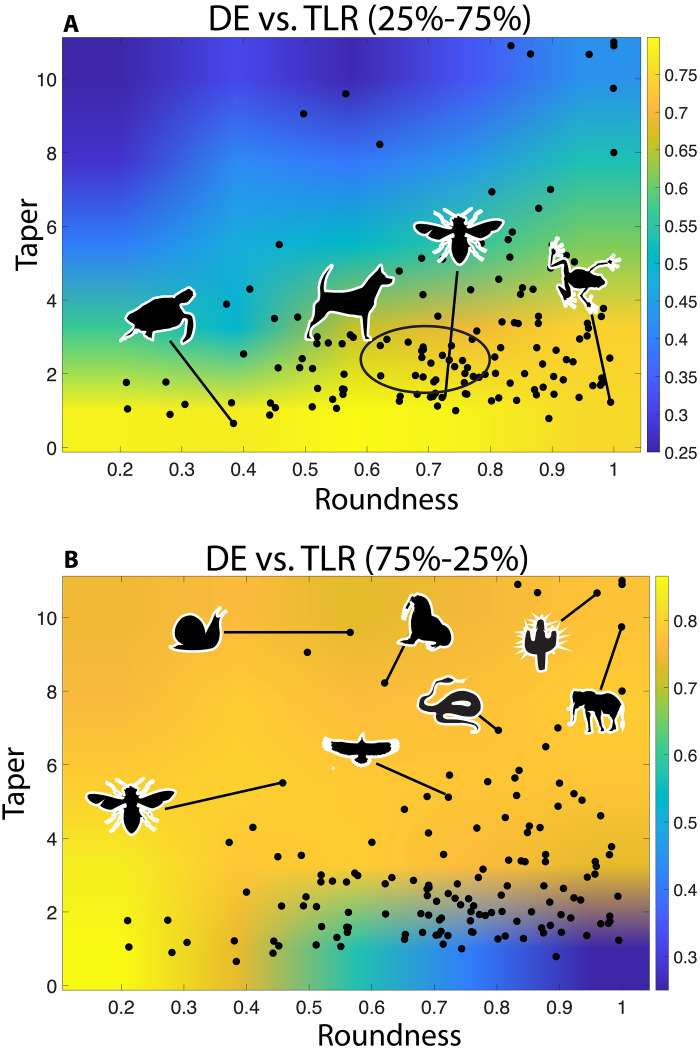
Combined performance landscapes of puncture tool mechanics show shifts in high performance when weighting between buckling resistance and energy efficiency is uneven. (**A**) Combined performance landscape when puncture efficiency (DE) is more heavily weighted than buckling resistance (TLR). (**B**) Combined performance landscape when buckling resistance (TLR) is more heavily weighted than puncture efficiency (DE). Silhouettes in both figures illustrate which types of organisms fall in high-performance regions depending on the weighting schema.

As expected, when combined landscapes are created that weight either puncture efficiency or buckling more highly than the other, the resulting landscapes begin to shift toward the patterns seen in the individual performance landscapes: high performance for slender, flatter tools when puncture efficiency is weighted more and high performance for squatter, more round tools when buckling resistance is weighted more highly (Fig. 4).

### Puncture tool diversity

A large majority of morphological data from 143 biological puncture tools (including vertebrates, invertebrates, and plants) clusters along the region of high performance seen in the combined performance landscape where buckling resistance and puncture efficiency are equally weighted (Figs. 2 and 3). Of these, a small set of diverse species falls directly on the thin ridge of highest performance including scorpion stingers, dracula ant mandibles, peccary tusks, hoopoe beaks, mantis shrimp appendages, and thorns from the Bull’s Horn plant. A larger diversity falls just off this high-performance ridge, into regions of lower performance in the equally weighted landscape. However, these regions of morphospace become high-performance peaks when buckling resistance and puncture efficiency are weighted unevenly (Fig. 4).

When puncture efficiency is weighted more importantly (Fig. 4B), the highest performance region encompasses far more area with a spread of biological examples including raptor claws, snake fangs, trap-jaw ant mandibles, woodpecker beaks, and parasitoid wasp ovipositors. This region of high performance also includes several extreme outliers with very high taper and high roundness such as defensive spines found on cacti. Their placement may be due to being disposable features, where resistance to failure is less biologically relevant (see below). Another set of species fall in an otherwise unoccupied space: high taper and mid-range roundness. These are a walrus tusk, catfish spine, and the love dart of the Catalina cactus snail. In contrast, when buckling resistance is weighted more heavily (Fig. 4A), the high-performance region shifts to low taper and high roundness and covers a large portion of taxa including mammalian canines, raptor beaks, crocodylomorph teeth, and even theropod teeth.

A final region of interest falls in the lower left corner of the plot, squat, flat, almost blade-like tools that seem to be in a region of high performance regardless of how buckling resistance and puncture performance are weighted. These include shark and barracuda teeth as well as examples of cone snail harpoons and land snail love darts. Why all biological forms do not occupy this region, or at least not more of them, is likely due to other factors involved in puncture function as outlined below.

These identified patterns should not be misconstrued with specific evolutionary patterns, as we are not examining changes in trait states over time or across phylogenetic hypotheses. They do, however, reveal potential widespread convergence in shape and structure across kingdoms keyed into specific puncture mechanics.

### Taxonomic affinity

While the species included in this study are too diverse (spanning multiple phyla) to use statistical comparative methods to test for phylogenetic signal, we can perform a qualitative survey of how related taxa plot in relation to each other. Figures 2 and 3 illustrate a couple interesting examples. Some taxonomic groups cluster fairly tightly, such as mammalian carnivorans (although note that walrus tusks are an exception to this pattern). In contrast, certain groups spread across a wide area of the morphospace. The four parasitoid wasp species included encompass more space than all of the carnivorans combined. One potential explanation is that with a few exceptions (bears, walrus, etc.), most carnivorans primarily consume meat, whereas parasitoid wasps are extremely diverse (one of the most specious clades known), given the vast diversity of host substrates (*44*). We use a single generalized model material to evaluate puncture performance, which may result in the vastly different performance between species of wasps whose tools evolved to puncture very different substrate materials. Less clear, however, is the vast diversity seen in love darts, calcified tools used by hermaphroditic snails during mating (*9*). These show broad diversity, covering almost the entire range of biological tools, yet are all used for puncturing other snails.

**Fig. 5.**
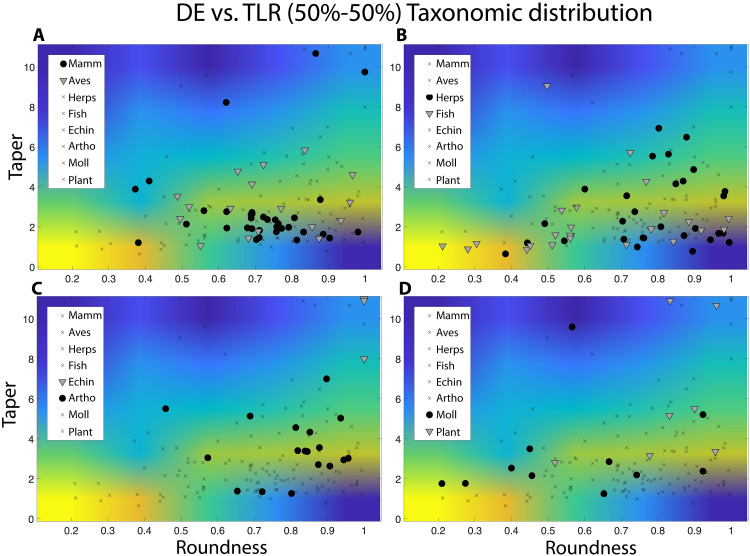
Mapping taxonomic groupings of biological puncture tools onto the combined performance landscape shows limited taxonomic patterns. (**A**) Plot with mammals and aves highlighted. (**B**) Plot with herps (archosaurs, squamates, turtles, and amphibians) and fish highlighted. (**C**) Plot with echinoderms (three sea urchins) and arthropods highlighted. (**D**) Plot with molluscs and plants highlighted. These plots illustrate that while there may be some constraints in certain clades, there is no overall taxonomic pattern to the biological data presented here.

To get a better overall sense of how different phyla plot across our landscape, we labeled each puncture tool based on one of eight taxonomic categories: mammals, birds, herps (archosaurs, squamates, turtles, and amphibians), fish, echinoderms, arthropods, mollusks, and plants. Figure 5 illustrates how each of these categories is spread across the landscape. While there is an uneven sampling among these groups, it is clear that most of them show a widespread distribution across the space and a lot of overlap in distributions. This suggests that at least at a broad level, there is little taxonomic structure to the distribution. What does arise is that there may be some taxonomic constraints to the distribution. For instance, the lower left corner (low taper and low roundness) is occupied solely by fish and mollusks, while the upper right (high taper and high roundness) is occupied by mammals, echinoderms (sea urchins), and plants. However, these patterns may be due to sampling, and a broader survey is required to verify whether these represent potentially real constraints.

### Functional behavior

**Fig. 6.**
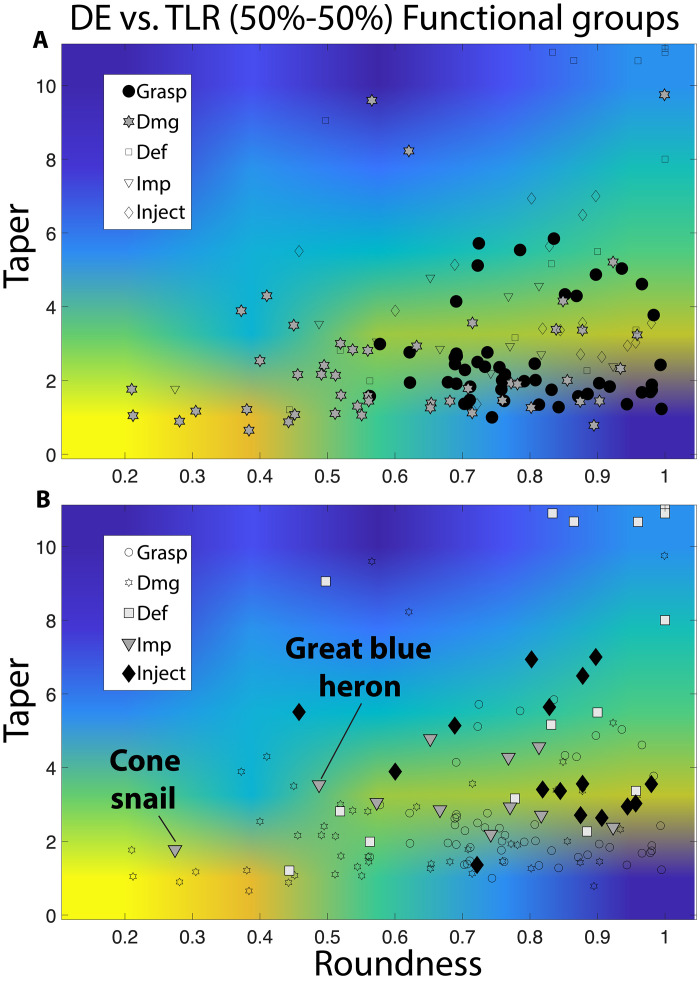
Mapping of potential function of biological puncture tools onto the combined performance landscape offers insights into biological tool diversity. The biological data plotted onto the evenly weighted combined performance landscape are grouped into five broad categories of potential puncture function. (**A**) Graspers and damage dealers are highlighted. (**B**) Defensive, impaling, and injecting spines are highlighted. Certain groups, such as injectors and graspers, show somewhat restricted space occupation, while damage dealers show the widest spread of examples. Impaling forms are also restricted with the exception of the great blue heron and the ivory cone snail, both highlighted in (B).

Not all puncture tools are used for the same biological function. Organisms use puncture tools to capture prey, to inject substances, as defense mechanisms or just to cause damage (*3*). Beyond the buckling resistance/puncture efficiency trade-off, these different functions may constrain morphology of the tools. To examine this, we assigned each tool to one of five generalized functional groups: Impaling tools are used to puncture and then remain attached to the target, like a harpoon. Examples include spearing mantis shrimp claws or the bills of some birds. Grasping forms use multiple tools moving in opposition to puncture and restrain a target. Examples include carnivoran canines and several types of insect mandibles. Injection tools use an internal tube to introduce a fluid or solid object to a target. Examples include venomous snake fangs and parasitoid wasps. Damage tools have the primary function of creating as much fracture/damage as possible. This can be simply to wound the target as in some sharks or to create more surface to introduce substances such as the diverse shapes of love darts. Last, defensive tools are used to simply deter aggressive organisms and may not need to fully puncture. Examples include cactus spines or porcupine quills. These assignments are plotted in Fig. 6 and show different levels of tool shape diversity. Grasping, injecting, and harpooning have more restricted ranges, while defense and especially damage fill almost the entire biological range.

The restricted range for injecting tools makes sense in light of what this function requires. Injectors must have space within their structure for the fluid (or solid in the case of an egg) to move through during injection. It is expected that no injectors are found in the left side of the plot, where tools tend to be flattened, without such space. They are also not found at either very low or very high taper values, favoring a middle range on that axis. These tools require a high puncture efficiency but are constrained by the need to resist buckling confounded by the presence of a tube or opening for the substance being injected to move through.

Both graspers and impalers have similarly restricted ranges, overlapping with injectors to an extent but falling lower on the taper axis (Fig. 6). These tools are generally circular in their shape, pointing to evolution toward robustness, something borne-out when compared with the combined landscapes in Figs. 2 to 4. The graspers in particular fall in a region that shows up as high performance when buckling resistance is more heavily weighted. While the impalers mostly overlap with graspers, there are two exceptions of impalers that spread farther along the roundness axis. The first is the great blue heron, which uses its beak to harpoon fishes and is slightly more flattened than other impaling tools. However, the real outlier is the ivory cone, a cone snail which uses its harpoon to capture prey. This species is much flatter than the other cone snails included in this study, and it is uncertain why it has evolved such a shape.

By far, the most diverse functional category is damage with defense close to it. Damaging tools make up almost all of the tools falling below 0.5 roundness (with a few exceptions). Flatter tools, while not potentially as useful for grasping or injecting, make solid blades for cutting and creating fracture surfaces and damage. However, damage dealing tools are also found across other regions of occupied space illustrating that dealing damage may not place as many physical constraints on tool design, although the target material likely does. Defensive tools are almost as wide ranging in form, but fewer of them show a flattened shape. Since defensive tools tend to be passive, the organism has little control over what direction the incoming load is at; therefore, a more circular cross section may ensure equal resistance to bending in any direction.

It should be noted that these definitions of function are artificial to an extent, as some puncture tools may be used for a variety of functions. As an example, carnivoran canines are almost universally categorized as grasping here, which would imply that they are only used for holding prey. This ignores the very real possibility of these teeth being used for more general damage dealing. We opted to categorize them as graspers as even when applying damage, they likely use both opposing sets of tools for a bite. Exceptions to this, such as Smilodon or walrus that use isolated enlarged fangs to puncture, are categorized as damage dealers. While this point can be argued, it is notable that changing canines to damage dealers would have little effect on the patterns observed in Fig. 6.

### Disposability

**Fig. 7.**
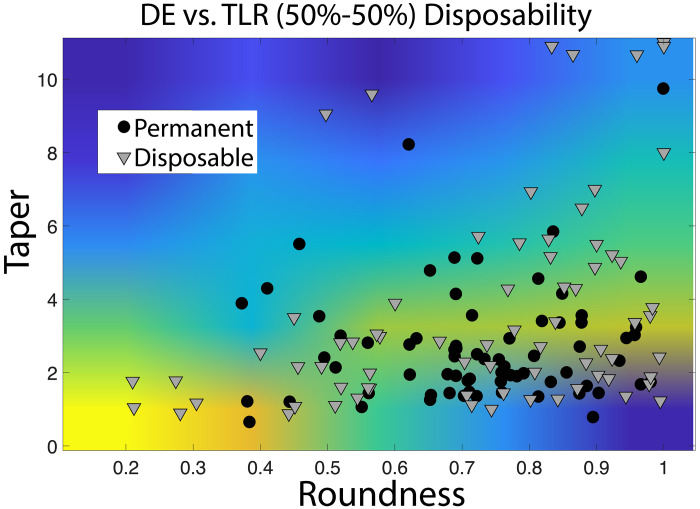
Mapping of the disposability of biological puncture tools onto the combined performance landscape offers insights into biological tool diversity. Biological data highlighted as either permanent or disposable/replaceable tools. While there is not much pattern to be seen between these categories, the disposable tools do occupy a slightly larger range and most of the extreme forms.

Not all puncture tools are built to last a lifetime. In many organisms, the puncture tool is replaceable if it gets broken, like in mantis shrimp which can regrow their spearing appendages during molting. However, this can take time and a number of molt cycles. Other puncture tools are completely disposable: Shark jaws often have multiple rows of teeth in reserve, and cone snail harpoons are meant to be used only once and even the mandibles or stingers of colonial ants, where the individual workers themselves are disposable in service to the colony. We might expect tools that are disposable (or just easily replaceable) to favor puncture efficiency over buckling resistance and occupy a different region of tool space relative to more permanent tools. However, we find the opposite (Fig. 7). There is no real separation between different levels of replaceability. This may be due to the fact that when TLR is weighted lower, the area of high-performance expands, meaning that there would be little evolutionary pressure among the disposable forms in any particular direction. Furthermore, while permanent tools show a slightly more restricted space occupation, there are outliers. Both walrus and elephant tusks show very high taper values, which might be assumed to make them fragile and prone to breakage. Some of this may be explained by differences in materials as the buckling resistance is only partly controlled by structure; stronger materials likely allow for thinner tools to still be resilient enough to be permanent.

## DISCUSSION

The combined performance landscape illustrates how reducing puncture tool geometry to just a few key traits (taper and roundness) can offer real insights into puncture tool diversity across biology. There is always a balance when making models of biological form or processes between simplifying the morphology enough to be feasibly analyzed yet including enough complexity to allow insight to biological reality (*45*). Work on puncture tool diversity has often focused on just one aspect of tool shape; slenderness (taper here). However, recent work on puncture mechanics has illustrated the importance of cross-sectional shape (*15*, *34*). Our landscapes here use both taper and roundness to describe tool shape and place them into a mechanical context. However, there are still more aspects of shape that likely have strong influence on functional morphology, and the patterns we describe here must be considered in light of them as well.

We treated our puncture tools as straight-edged cones, which are a simplification of actual biological puncture structures. Notably, actual straight-edged cones would come to an infinite point at their apex, something that is impossible in nature. In biological forms, it is necessarily the case that near the tip of these puncture tools the sides must curve to make a more rounded tip. Effectively, measures of taper (height versus cross-sectional width; see Materials and Methods) near the tip will be lower than those taken at the base, due to the sides needing to curve toward each other as the tip becomes more rounded. This aspect of geometry in elongate pointed biological structures has been explored in recent papers that suggest that the overall shape is better described by power laws (*38*, *39*), which can account for these changes in the slope of the sides along the tool length.

In this study, we took the taper measures from the base of the biological puncture tools’ working distance, meaning that all of our measures are likely overestimates of the taper near the tip, depending on the specific power slope a tool follows (*37*, *38*). Our digital models also assumed a constant taper with rounded the tips as discussed in Materials and Methods. This assumption may influence aspects of our performance results. It should be noted that the biggest changes will be near the tips of these tools, although work on power curves has shown that certain tools may show deviate further down as well (*37*, *38*). Exactly how much this aspect of tool shape would influence the performance results is unclear. Our analyses here act as a good baseline for exploring the effects of these more nuanced shape parameters in future studies.

Many biological puncture tools are curved, from the slight curve found in canines up to the half-moon shapes found in certain avian claws. It is intuitive that curvature may play a role in the performance of puncture tools; however, previous experimental and modeling work has shown that curvature likely does not influence puncture efficiency (*46*). These studies argue that curved tools likely relate to rotational motion of the tool as it punctures, since most puncturing tools are actuated by elements rotating around joints (teeth, claws, etc.). Buckling resistance is likely to be influenced by tool curvature, as has been shown in previous work (*21*, *47*). We speculate that all else being equal, increasing the curvature of a puncture tool weakens its buckling resistance, potentially creating an evolutionary pressure to move toward lower taper, but this needs to be tested.

Another key aspect of tool shape that is not incorporated here is surface ornamentation/surface roughness. One of the key aspects of puncture mechanics that has been highlighted in previous work is friction created by the puncture tool moving into the target. The energy required to overcome this friction has been shown to be a key component of the energetics of the system, directly correlated to the volume of the tool (*34*). Even the shape variance seen in this study could influence friction, as the cone models were scaled to have identical length, which will lead to variation in volume. Cones with lower taper will have larger volume, while flatter cones will have smaller volume. Beyond just this variation in volume, however, many organisms have ornamentation which can alter this frictional component of puncture mechanics. Many teeth have serrated edges or bladed edges along the length. This type of ornamentation can reduce the contact area between the tool and the material it is puncturing, reducing overall friction relative to a similar tool with a smooth surface (*6*). Other types of ornamentation, like the hook structures on cone snail harpoons, catch onto materials during puncture making the tools hard to remove. It has been shown in both porcupine quills and cacti that the same ornamentation can make puncturing easier while making removal of the tool much harder (*6*, *7*). All of these can influence the result found here and make the mechanics of puncture tools more complex.

Beyond just tool shape, there are other variables associated with puncture systems that will influence shape diversity, either by creating more constraints or allowing evolution to circumvent existing constraints. The material properties of both the tool and the target material will have a strong influence on what shapes are most effective. Differences in material properties of puncture tools may help explain some of the distribution across the performance optima. Different structures and taxa have different materials, which allow or necessitate specific limits on roundness or taper. As noted above, elephants and walrus can potentially maintain more slender forms in their tusks by having stronger materials (enamel/dentine). In other cases, the tools may actually be designed to break or at least separate from the animal to allow for escape such as in porcupine quills. The composition of the materials being targeted for puncture can also alter aspects of puncture performance, especially as most biological materials systems are composites with different layered materials that can have varying properties (*48*). In particular, even a relatively thin integument has been shown to significantly decrease puncture efficiency (*26*), potentially requiring shifts in tool morphology. The materials of the tool will also influence how much the tool may wear over a lifetime of use. Wear over time has been shown to affect puncture performance in teeth (*14*). This will specifically influence permanent tools that cannot be replaced.

The combination of combined performance landscapes based on puncture energetics with biological data presented here illustrates the power of using simplified geometric rules and measures to describe morphological diversity. By connecting morphology based on two simple parameters, taper and roundness, with the emergent mechanical performance, buckling resistance, and puncture efficiency, we are able to simultaneously identify unifying trends in biological puncture diversity while still recognizing the outliers that hint at a greater complexity. Our landscape not only identifies the trade-offs between buckling resistance and puncture efficiency as well as between fracture creation and material deformation but also shows how these trade-offs may directly relate to the diversity of puncture tool shape. Outlier biological forms, acting as exceptions to these trends, offer additional insight into how factors such as materials, function, and dynamics may be further influencing diversity. Together, the use of simplified morphological forms to connect mechanical performance estimates to the actual range of biological diversity allows for greater insights into the relationship between form and function across biology and reveals how complex the evolution of biomechanical systems can be, even when they are seemingly simple such as puncture tools.

## MATERIALS AND METHODS

### Theoretical morphospace

We constructed a theoretical morphospace of puncture shape based on two shape traits: taper and roundness. Taper is measured as the height of the puncture tool divided by the width of its cross section. Taper ranges from “0” to “infinite” (the higher the value, the more slender the tool is). Roundness is defined as the ratio between the minor and major axes of the cross section at its base. This trait ranges from 0 to 1, with 1 being a perfectly circular cross section (high roundness), with the cross section becoming more and more elliptical as it moves toward 0 (low roundness). For tools where roundness is less than 1, the major axis of the cross section is used as the width for taper measures, resulting in a “max” taper for such tools.

To evaluate performance across this morphospace, we defined 25 distinct cone morphologies based on unique combinations of taper (2, 4, 6, 8, and 10) and roundness (0.2, 0.4, 0.6, 0.8, and 1.0). For the comparative FEAs, all cones were scaled to have the same length/height. We chose length as our standard because it allows for straightforward design for the puncture FEA described below: All puncture tests are performed to the same overall depth of puncture. Standardizing by length does mean that surface area and volume will vary between cone models, which will have an impact on FEA results (*49*). However, this variation is biologically relevant, as area influences surface creation and volume will alter deformation. As this variation is what we are specifically interested in exploring, we do not want to eliminate them from the analysis by standardizing by either area or volume.

Each of 25 modeled cones was tested for both their resistance to buckling under tip loading both parallel (TLR) and perpendicular (SLR-E and SLR-F) to the long axis of the cone. These same models were used to test for puncture efficiency using two metrics: energy required to create fracture surface (FE) and energy lost due to target material deformation (DE).

### Buckling resistance analysis

FEA of the resistance to bending and buckling of modeled puncture tools was performed in Abaqus. Cones with prescribed taper and roundness were modeled in Solidworks and imported into Abaqus. The cones were given a standardized height of 15.05 mm. The cones were rounded with semispherical tips with diameters equal to 0.15 mm or ~1% of the standard height. These cones were then assigned material properties comparable to enamel (Young’s modulus = 80 GPa, Poisson’s ratio = 0.3). The measured material properties of enamel can actually vary widely (*50*). Since the numerical results are less important than the comparative values on the scaled landscapes, we use values for both properties we have previously used and that fall within the range of published values (*51*–*53*). The cone base was assigned “encastre” (fully fixed) boundary conditions. A uniaxial traction load of 10 N/mm^2^ was applied to the entire rounded tip (112 nodes total), with three orthogonal loading directions tested for each cone: the negative *y* direction (−*y*) (along the central axis), the positive *z* direction (+*z*), and the positive *x* direction (+*x*) (both perpendicular to the central axis of the cone). Four-node tetrahedral elements (C3D4) were used to generate a gradient mesh toward the tip region, with an average seed size of ~0.1 mm. To evaluate the effect of applied loads, the total stored strain energy (ALLSE) was extracted from the field output and compared across cone shapes. Lower ALLSE values correspond to bending/buckling-resistant cone shapes. TLR and SLR are based on strain energy (ALLSE), and the values of which are reported in data S1.

### Puncture efficiency analysis

Puncture efficiency of prescribed cone shapes was evaluated in Abaqus Dynamic/Explicit following a previously established framework for simulating puncture-induced damage and associated energetics in soft tissues (*34*). As shown in fig. S1, a cone and a cylindrical half-space substrate were aligned to form an axisymmetric contact pair. The substrate was modeled as a generalized incompressible neo-Hookean hyperelastic material (normalized shear modulus = 1, Poisson’s ratio ≈ 0.5). For simplicity, the cone was modeled as a rigid, impenetrable surface due to the order-of-magnitude difference in stiffness between the cone and substrate materials. All other elastic material properties were normalized by the shear modulus of the substrate in the final analysis. Gradient meshing was applied to both the substrate and cone near the contact interface. Eight-node brick elements (C3D8R) and six-node wedge elements (C3D6) were assigned to the substrate depending on location, while three-node rigid elements (R3D3) were used for the cone. The interaction between the cone and the substrate adopted a frictionless contact. We used the frictionless condition for three reasons: (i) Frictional energy is not the focus of the puncture performance analysis; (ii) we assume that the effect of friction is minor for a slender tool and small depth of puncture. (iii) Simulations with friction are challenging and can lead to unconverged results and unexpected singularities.

During each puncture simulation, the cone advanced into the substrate along the central axis at a constant dynamic speed of 0.5 m/s until reaching the maximum displacement of *d* = 5 mm. Puncture damage was modeled via element deletion based on a critical strain energy failure criterion implemented through a user-defined field (VUSDFLD), as detailed by Zhang and Anderson (*34*). Three normalized energetic metrics of puncture performance were calculated from the full-field integration of elemental energy components: stored strain energy (Δ*U*_e_*/*μ*d^3^*), work to fracture (*W/*μ*d*^3^), and puncture coefficient of efficiency [*W*/(*W* + Δ*U*_e_)], where μ is the shear modulus of the substrate. Stored strain energy is used for DE, work to fracture is used for FE, and both are reported in data S1.

### Performance landscape

Results from the buckling resistance and puncture efficiency analyses are used to create performance landscapes over the theoretical morphospace of puncture tools. Because the various performance measures (TLR, SLR, FE, and DE) have such different scales, the first step is to normalize them onto the same scale. The performance data were normalized using the following equation

$$\text{Normalized value}=1-\frac{\text{Original value}-\text{Min}}{\text{Max}-\text{Min}}$$

This results in each performance metric having a range from 0 to 1, with 1 being “high performance” and 0 being “low performance.” Once the data have been scaled, and individual performance landscapes are created in MATLAB for each performance metric using the data from the 25 cones that are evenly spread across the space. We used the interpolation feature of the Imagesc command to create the performance heatmaps. We specifically used a bilinear method where the value of a given pixel located at (*x*, *y*) is a weighted average of the surrounding pixels. On all landscapes, yellow indicates high performance, and blue indicates low performance.

We created combined landscapes following the method of Stayton (*43*). When combining performance landscapes, the various performance metrics must be weighted in terms of how important they are assumed to be for that landscape. Each trait is multiplied by a weighting factor, with all weighting factors summing to 1. As an example, if we want to combine DE and TLR landscapes and have them equally weighted, we take each theoretical tool shape and multiply its scaled values of TLR and DE by 0.5 and then sum the results to obtain the new combined performance value for that shape. Calculating these new performance values for the shapes and running the interpolation in MATLAB gives a combined performance landscape. We examined each pairwise comparison between buckling and puncture performance.

### Biodiversity data

To compare our theoretical spaces to actual biological puncture tool diversity, we collected measures of taper and roundness from 143 different biological puncture tools representing 133 different species across multiple phyla. The dataset includes all major groups such as vertebrates (mammals, aves, reptiles, amphibians, fish, and stem gnathostomes), invertebrates (arthropods, molluscs, etc.), and even plants. These specimens were sourced from a combination of museum and personal collections, three-dimensional scans made available via Morphosource, and, in some cases, directly from the literature when sufficient views of the tools allow for roundness to be measured (see data S2 for full citations).

**Fig. 8.**
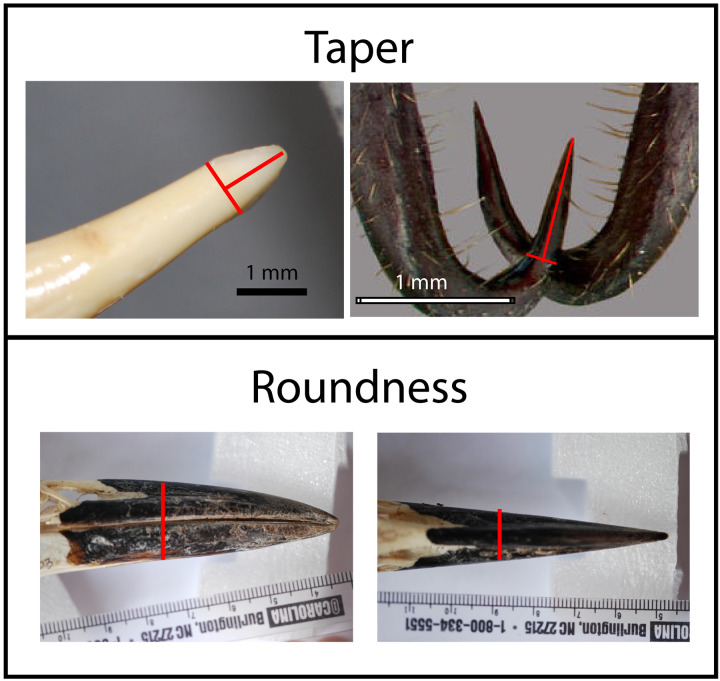
Examples of biological data measurements. (**A**) Two examples of measures of taper on a gar tooth (left; FMNH51333; photo by P.S.L.A.) and an army ant mandible [right; CASENT0009221; photo by A. Nobile; antweb.org (*55*)] under a CC BY 4.0 license (https://creativecommons.org/licenses/by/4.0/). (**B**) Example of roundness being measured on a night heron (FMNH436403; photo by P. Anderson).

For the taper measures, we had two criteria for determining the cutoff for the maximum width measure. The first was where there is a clear change in taper, as illustrated in Fig. 8A where the gar tooth has a taper at the distal portion of the tooth that abruptly ends, becoming almost untapered at a certain point. In cases where the taper never seems to change, we would test taper measures at multiple points along the length to verify this. The second is if the taper never seems to end, but there is an obvious point where the puncture insertion would end, as shown in Fig. 8B with the army ant mandible, which has an almost 180° curve along its length. Figure 8C also shows an example of a roundness measurement from a great blue heron beak. The raw measures can be found in the Supplementary Materials.

We attempted to cover a broad range of puncture tool shapes in this dataset, with a range of taper values from approximately 1 to 10 and roundness values from 0.2 to 1. While this encompasses a good range of the diversity of biological puncture tools, it likely misses some. Part of the difficulty is establishing what morphological elements that are long and slender actually function as puncture tools. In some taxa, such as fish, elongate spines may not be primarily used for puncture as much as simply making the organism too large to be swallowed. Recent papers on dental diversity show that teeth in particular can achieve values of taper less than one (*54*). However, at some point, these tools are no longer piercing and inserting themselves like puncture tools and are instead acting as blunt tools for crushing. Where exactly that cutoff would be on the taper scale or if there even is a discrete boundary is questionable. For the sake of this study, we chose an arbitrary cutoff that still encompasses a lot of diversity.

The taxa measured were assigned three types of categorical values: taxonomic group, tool function, and disposability. For the taxonomic group, we assigned tools based on high-level taxonomy to one of eight phyla categories: mammals, including several extinct sabertoothed forms; aves; herps, made up of archosaurs (including a nonavian dinosaur), squamates, turtles, and amphibians; fish, including the stem gnathostome *Dunkleosteus*; echinoderms, made entirely of sea urchin spines; arthropods, consisting here of mostly insects with a couple arachnids and one crustacean; mollusks, including both cone snails and love darts; and plants. The sampling across these groups is uneven, and the patterns seen should be considered preliminary pending greater sampling.

For the tool function, assumptions were made on the basis of the most common use of the tool as detailed here in the main text. Briefly, grasping tools must be set opposed to another tool, creating a situation similar to a vice; impaling tools are single tools that harpoon/capture prey without the aid of opposing tools; injecting tools specifically pierce and then inject a substance such as toxins or eggs; damaging tools are those obviously meant to cause damage but without any obvious other role such as gripping or injection; and last, defense tools are a catch-all for passive puncture tools that likely only puncture via forces created by the target as it attempts to attack the taxon with the tool. These are necessarily broad definitions and likely several could be applied to certain tools, but the purpose of this category is not to statistically differentiate them but to explore the potential ranges of different tool functions.

Disposability is a binary categorical variable based on simply whether the animal can replace the puncture tool in case it is broken. In our study “disposable” includes animals that can repair broken tools via molting (such as in mantis shrimp and other arthropods) as well as those that simply create so many tools that the loss of one does not matter (cactus spines and shark teeth). Note that mammalian teeth, which are replaced once during growth, are considered “permanent” for our purposes, since that replacement can only occur once.
