# Supplementary material for: Trade-offs in mechanical performance influence the diversity of fangs, stingers, and spines
Source: Sci Adv. 2026 Jul 8;12(28):eaec5395. doi: 10.1126/sciadv.aec5395 (PMC13344252; doi:10.1126/sciadv.aec5395)
Supplement: Supplementary file 1 — Fig. S1 Legends for data S1 and S2 [file sciadv.aec5395_sm.pdf]

Science Advances
AAAS

Supplementary Materials for

Trade-offs in mechanical performance influence the diversity of fangs, stingers, and spines

Philip S. L. Anderson *et al.*

Corresponding author: Philip S. L. Anderson, andersps@illinois.edu

Sci. Adv. **12**, eaec5395 (2026)
DOI: 10.1126/sciadv.aec5395

The PDF file includes:

Fig. S1
Legends for data S1 and S2

Other Supplementary Material for this manuscript includes the following:

Data S1 and S2

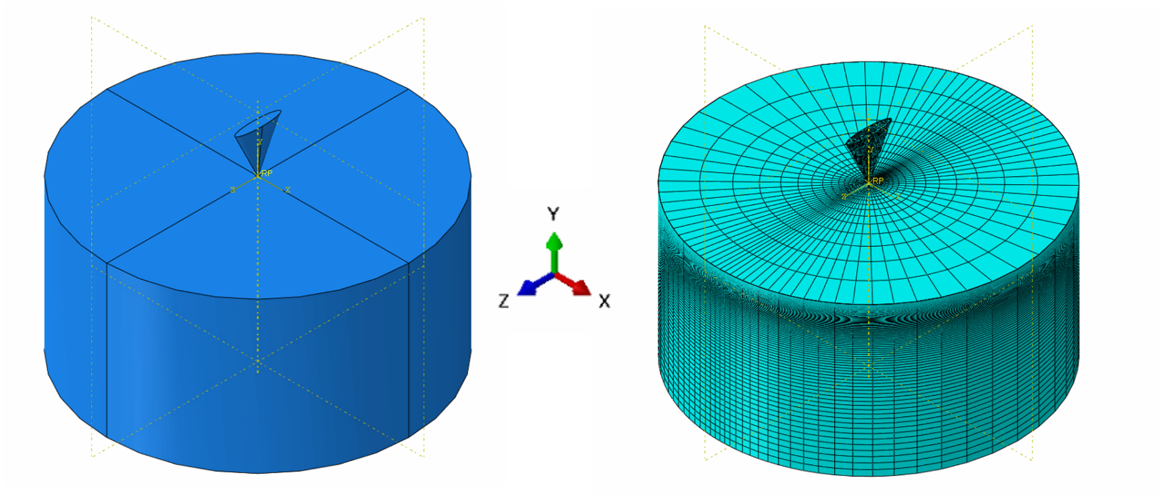

Fig. S1: **Puncture efficiency FEA design.** The cones are aligned with a cylindrical half-space substrate to form an axisymmetric contact pair. Gradient meshing was applied to both the substrate and cone near the contact interface. Eight-node brick elements (C3D8R) and six-node wedge elements (C3D6) were assigned to the substrate depending on location, while three-node rigid elements (R3D3) were used for the cone. During each puncture simulation, the cone advanced into the substrate along the central axis at a constant dynamic speed of 0.5 m/s until reaching the maximum displacement of d = 5 mm.

Data S1. (separate file)

Performance landscape data used to create the gradients in the landscapes.

Data S2. (separate file)

Biological measurements for 143 species used to plot the species in the performance landscapes.
